# Dr. Levi J. Hixon

**Published:** 1903-01

**Authors:** 


DR. LEVI J. HIXON, of La Salle, N. Y., died suddenly at his home December 5, 1902, from the effects, it was thought, of an overdose of medicine which he had taken inadvertently. He was a native of Canada and resided at La Salle about 18 years. He is survived by a widow and a young daughter.

